# Developmental succession of the microbiome of *Culex* mosquitoes

**DOI:** 10.1186/s12866-015-0475-8

**Published:** 2015-07-24

**Authors:** Dagne Duguma, Michael W. Hall, Paul Rugman-Jones, Richard Stouthamer, Olle Terenius, Josh D. Neufeld, William E. Walton

**Affiliations:** Department of Entomology, University of California Riverside, Riverside, CA 92521 USA; Department of Biology, University of Waterloo, Waterloo, Ontario N2L 3G1 Canada; Department of Ecology, Swedish University of Agricultural Sciences (SLU), Uppsala, Sweden; Present address: Florida Medical Entomology Laboratory, University of Florida, Vero Beach, FL 32962 USA

**Keywords:** *Thorsellia*, Outdoor mesocosms, Bacteria, Biopesticide, Transstadial transmission

## Abstract

**Background:**

The native microflora associated with mosquitoes have important roles in mosquito development and vector competence. Sequencing of bacterial V3 region from 16S rRNA genes across the developmental stages of *Culex* mosquitoes (early and late larval instars, pupae and adults) was used to test the hypothesis that bacteria found in the larval stage of *Culex* are transstadially transmitted to the adult stage, and to compare the microbiomes of field-collected versus laboratory-reared mosquitoes.

**Results:**

Beta diversity analysis revealed that bacterial community structure differed among three life stages (larvae, pupae and adults) of *Culex tarsalis*. Although only ~2 % of the total number of bacterial OTUs were found in all stages, sequences from these OTUs accounted for nearly 82 % of the total bacterial sequences recovered from all stages. *Thorsellia* (*Gammaproteobacteria*) was the most abundant bacterial taxon found across all developmental stages of field-collected *Culex* mosquitoes, but was rare in mosquitoes from laboratory-reared colonies. The proportion of *Thorsellia* sequences in the microbiomes of mosquito life stages varied ontogenetically with the greatest proportions recovered from the pupae of *C. tarsalis* and the lowest from newly emerged adults. The microbiome of field-collected late instar larvae was not influenced significantly by differences in the microbiota of the habitat due to habitat age or biopesticide treatments. The microbiome diversity was the greatest in the early instar larvae and the lowest in laboratory-reared mosquitoes.

**Conclusions:**

Bacterial communities in early instar *C. tarsalis* larvae were significantly more diverse when compared to late instar larvae, pupae and newly emerged adults. Some of the bacterial OTUs found in the early instar larvae were also found across developmental stages. *Thorsellia* dominated the bacterial communities in field-collected immature stages but occurred at much lower relative abundance in adults. Differences in microbiota observed in larval habitats did not influence bacterial community profiles of late instar larvae or adults. However, bacterial communities in laboratory-reared *C. tarsalis* larvae differed significantly from the field. Determining the role of *Thorsellia* in mosquitoes and its distribution across different species of mosquitoes warrants further investigation.

**Electronic supplementary material:**

The online version of this article (doi:10.1186/s12866-015-0475-8) contains supplementary material, which is available to authorized users.

## Background

Bacteria ingested by the immature stages of mosquitoes (Culicidae) are generally known to provide nutrition [1, 2], facilitate successful development [3, 4], and influence vector competence [5–8]. The presence of certain bacteria has been also shown experimentally to provide immunity against pathogens, and reduce vector competence [9–11]. Therefore, interest has grown in understanding the obligate and facultative roles of the gut microflora of mosquitoes, their interaction with parasites and their manipulation to devise alternative and more sustainable vector control strategies (e.g., paratransgenic control) [7, 9, 12–16].

To date, the majority of the studies on microbiomes of mosquitoes have focused largely on investigating the gut microflora (and/or symbionts) associated only with adult mosquitoes (e.g., 9). The focus on adult mosquitoes is justified, on one hand, because adult mosquitoes transmit pathogens directly or cause nuisance to humans and animals. On the other hand, the interactions of bacteria and immature stages of mosquitoes will potentially influence the microbiome of the adult mosquitoes. Moreover, previous studies focused on the dominant tropical disease vector mosquito species such as *Anopheles gambiae*, and not much was known about North American native mosquito species such as *Culex tarsalis.* Few studies have considered transstadial transmission (larvae to pupae to adult) of microbial communities because the entire midgut of culicid mosquitoes is generally thought be replaced during development such that entire bacterial communities associated with the larval midgut are eliminated prior to eclosion [2, 17]. However, circumstantial evidence in *Anopheles* mosquitoes suggested that some bacteria species found in larval stages persist through metamorphosis and are transferred to adults [18, 19]. Recently, these contradictory observations have been reconciled in studies from *Anopheles stephensi* where the Malpighian tubules function as a “refugium” for bacteria during metamorphosis [20, 21]. In addition, most other studies were based on laboratory-reared mosquitoes, fed standardized diets, and raised under controlled environmental conditions for several generations. However, these artificial conditions are likely to restrict and/or alter the host microbiomes of lab-raised insect populations relative to natural populations (e.g. *Drosophila*; [22]).

The Gram-negative genus *Thorsellia* (*Gammaproteobacteria*) is the most abundant bacterial group found among the gut microflora of field-collected late instar larvae of *Culex* spp. (3^rd^ and 4^th^ instars; [23]). *Thorsellia anophelis* was first described from adult *Anopheles arabiensis* in Kenya [24, 25] and is the type specimen for a new family of the *Bacteria*, *Thorselliaceae* [26]. *Thorsellia anophelis* has been reported to be the predominant bacterial species found in adult *Anopheles gambiae* sensu lato [27] and has also been found in *Anopheles culicifacies* [28]. It has been hypothesized that the bacterium is acquired during larval feeding, and is then transferred transstadially to adult *Anopheles*, although the evidence is somewhat conflicting [18, 19, 27]. Rani and colleagues recovered this bacterium in both larvae and adults of *Anopheles stephensi*, but not in the pupal stage [18], whereas Briones and colleagues did not recover the bacterium from either larval or pupal stages of *Anopheles* [27]. However, others found *Thorsellia* to be the 5^th^ most abundant genus in *Anopheles gambiae* larvae, and the 10^th^ most abundant genus in pupae [19]. Direct evidence supporting the transstadial transmission *of Thorsellia* and other bacterial species in the microbiome of *Culex* mosquitoes is lacking.

It was unknown whether succession or other factors such as use of pesticides causing changes in bacterial communities of the larval developmental sites also affect the microbiome in mosquitoes. Pesticides (i.e., malathion, permethrin, atrazine and glyphosate) alter bacterial communities in the larval environment [29]. Removal of mosquito larvae using biopesticides such as *Bacillus thuringiensis* subsp. *israelensis* (*Bti*) was reported to lessen grazing pressure on bacterial communities [30–32] and increase the diversity of bacteria in the habitats [33].

In this study, we: 1) identified bacteria found in the guts of different developmental stages of *C. tarsalis*; 2) compared the microbiomes of field-collected *C. tarsalis* larvae with laboratory-reared individuals; and 3) assessed the effects of habitat age and manipulating larval mosquito density (using *Bti* applications) on the microbial communities found in late-instar mosquito larvae.

## Results

### Bacterial taxa in developmental stages of *C. tarsalis*

**Fig. 1:**
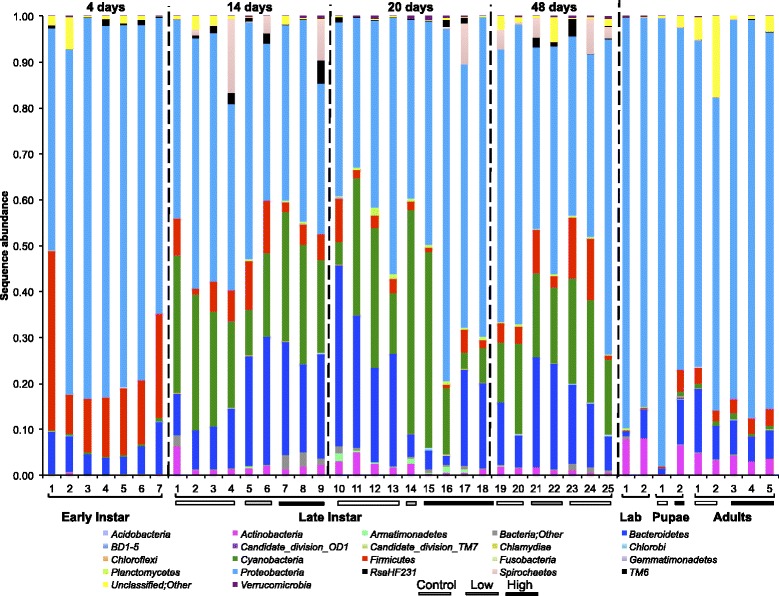
Proportional sequence abundance of bacterial phyla across *Culex* developmental stages. The samples were taken 4, 14, 20 and 48 days after the onset of the experiment. Only phyla with an average abundance ≥ 0.01 % were included. Numbers on the x-axis represent sample identification numbers. Stages of mosquitoes are denoted by Early instar, Late instar, Lab = lab reared late instar, Pupae, and Adults. Light bar refers to control; gray refers to low *Bti* and dark bar for high *Bti* treatments

A total of 14,634 OTUs (4,609,186 bacterial sequences) were generated from 41 *Culex* mosquito samples (39 field-collected and two laboratory-reared). *Proteobacteria* (56 %), *Bacteroidetes* (15 %), *Cyanobacteria* (14 %), *Firmicutes* (7 %), *Actinobacteria* (2 %), and *Spirochaetes* (2 %) were the most abundant bacterial phyla found in developmental stages of *Culex* mosquitoes (Additional file 1: Table S1). Unclassified sequences accounted for ~0.6 % of sequences, whereas, bacterial sequences unclassified to phyla accounted for 0.8 % of sequences. Another 30 phyla accounted for the remaining 1 % of the bacterial communities. Although the relative abundance of bacterial taxa changed across the mosquito developmental stages, *Proteobacteria* were the dominant bacteria found in the guts of *C. tarsalis* (Fig. 1).

**Fig. 2:**
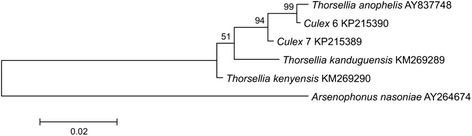
Molecular phylogenetic analysis of *Thorsellia* from *Culex* larvae by maximum likelihood method. The proportion of trees in which the associated taxa clustered together is shown next to the branches (1000 bootstraps). The tree is drawn to scale, with branch lengths measured in the number of substitutions per site

A total of 235 bacterial OTUs, comprising 82 % of the total bacterial sequences, were found among the three life stages of field-collected larvae (early and late instars), pupae, and adults. However, only 27 OTUs classified into seven phyla, representing 66 % of all the sequences recovered from the field-collected mosquitoes, were shared among the samples from all developmental stages (Additional file 2: Table S2). *Proteobacteria*, *Cyanobacteria, Bacteroidetes* and *Firmicutes* were the most abundant phyla shared among the different stages (Fig. 1). *Thorsellia* (40 %), *Cyanobacteria* (18.0 %) and *Dysgonomonas* (13 %) were the most abundant genera shared across developmental stages. A *Thorsellia* OTU (#2664) was found in all developmental stages of field-collected larvae, pupae, and adults of *C. tarsalis*, and was the most abundant bacterial species across all mosquito life stages (Additional file 2: Table S2). Phylogenetic analysis using longer 16S rRNA gene sequences (1070 bp) of *Thorsellia* obtained from *C. tarsalis* larvae revealed that this species is closely related to *T. anophelis* described from *Anopheles gambiae* sensu stricto but may also contain a separate strain (Fig. 2). The two *Thorsellia* sequences from *Culex* mosquitoes were both 99 % similar to *T. anophelis*, but with individual differences. Out of the six clones sequenced, five clones were identical to clone #6, whereas clone #7 was unique but had nucleotide differences that are supported by the other *Thorsellia* species (data not shown).

### Early instar larvae (not identified to species)

**Fig. 3:**
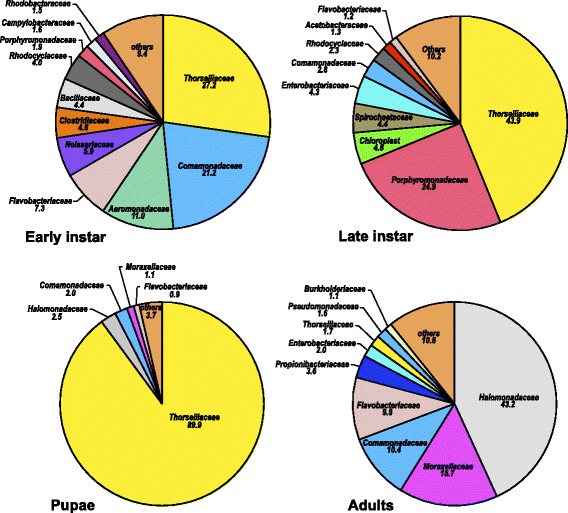
Family-level abundance of bacterial communities. Family-level abundance (%) of bacterial communities in field-collected early (1^st^ and 2^nd^) instar larvae, late (3^rd^ and 4^th^) instar larvae, pupae and adults of *C. tarsalis.* Only sequences classified to family level were included. Because the treatments effects on the gut bacterial community structure within each stage were not significantly different, mosquitoes from all treatments were included in this figure

A total of 5,888 OTUs (805,169 sequences from 7 samples) in 34 bacterial phyla were recovered from early (1^st^ and 2^nd^) instars of *Culex* larvae*. Proteobacteria* (73 %)*, Firmicutes* (18 %), and *Bacteroidetes* (7 %) dominated the early instar *Culex* larvae (Additional file 1: Table S1)*.* Overall, bacterial taxa in *Gammaproteobacteria* (43 %)*, Betaprotobacteria* (26 %) and *Bacilli* (*13 %*) were the three most abundant classes found in the early stages of the mosquito life cycle (Additional file 1: Table S1)*. Thorselliaceae* (27.2 %) and *Comamonadaceae* (21.2 %) were the two most abundant families found in early instar larvae (Fig. 3). *Thorsellia* was the most abundant (19 %) genus found in the early stages followed by an unclassified taxon of *Gammaproteobacteria* (8.8 %) and *Aeromonas* (7 %) (Additional file 3: Table S3).

### Late instar *C. tarsalis* larvae

#### Field-collected *C. tarsalis* larvae

Overall, 34 bacterial phyla (10,115 OTUs from 25 samples) were found in field-collected late larval (3^rd^ and 4^th^) instars. *Proteobacteria* accounted for nearly half (49 %) of the sequences followed by *Cyanobacteria* (20 %), *Bacteroidetes* (18 %), and *Firmicutes* (6 %) (Fig. 1). *Spirochaetes* and *Actinobacteria* accounted for nearly 3 % and 2 %, respectively, of the sequences recovered from late-instar larvae. *Thorselliaceae* (43.9 %) and *Porphyromonadaceae* (24.9 %) were the two most abundant families found in late-instar larvae (Fig. 2). *Thorsellia* was the most abundant genus in field-collected late instar larvae (27 %) followed by *Cyanobacteria* (19 %) and *Dysgonomonas* (*Bacteroidetes*; 11 %) (Additional file 4: Table S4).

#### Laboratory-reared larvae

**Fig. 4:**
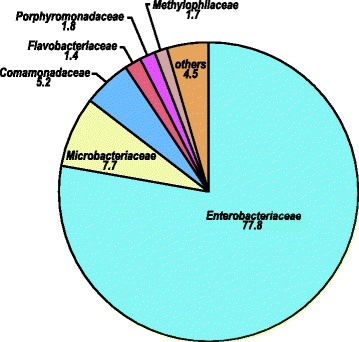
Family-level abundance (%) of bacterial communities in laboratory-reared late instar *C. tarsalis.* Only sequences classified to family level were included. These samples were from a laboratory *C. tarsalis* colony

The late-instar larvae from a laboratory colony were dominated by *Proteobacteria* (87 %) followed by *Actinobacteria* (8 %) and *Bacteroidetes* (4 %). Overall, 786 OTUs (319,786 sequences from 2 samples) were obtained from the laboratory-reared colonies. *Enterobacteriaceae* (77.8 %) was the most abundant family found in late instar laboratory-reared mosquitoes (Fig. 4). *Rahnella* (*Gammaproteobacteria*: *Enterobacteriaceae*) (64 %), unclassified *Enterobacteriaceae* (12 %), and unclassified *Microbacteriaceae* (*Actinobacteteria*) (8 %) dominated the bacterial communities in the larvae from the laboratory-reared colony (Additional file 5: Table S5).

In addition to the two OTUs of *Rahnella*, we found 24 other bacterial OTUs, including one OTU of each of *Thorsellia* (OTU# 2464), *Spirochaeta*, *Aeromonas*, *Enterobacteriaceae*, *Rhodocyclaceae*, *Bacillaes*, *Microbacteriaceae*, *Hydrogenophaga*, *Halomonadaceae*, *Alteromonadales*, *Flavobactrium*, *Diaphorobacter*, *Cloacibacterium*, *Aquabacterium*, *Propionibacterium*, *Sphingomonadales*, *Dysgomonas*, and two OTUs of *Cyanobacteria*, *Gammaproteobacteria* and *Clostridium* that were also shared among other samples.

In comparison to late instar larvae collected from the field, nearly 62 % (484/786) of all the OTUs found in lab were also found in the field-collected larvae, whereas only 5 % (484/10,115) of the OTUs found in the field-collected larvae were found in the lab colony.

### *C. tarsalis* pupae

Twenty phyla (represented by 638 OTUs from two samples) were recovered from *C. tarsalis* pupae among which *Proteobacteria* (96 %), *Bacteroidetes* (1.8 %), *Actinobacteria* (0.8 %), *Firmicutes* (0.7 %), and *Cyanobacteria* (0.1 %) were the dominant phyla (Additional file 1: Table S1). Unclassified bacteria accounted for 0.7 % of sequences. *Thorselliaceae* dominated the bacterial community at the family level (Fig. 3). Members of *Gammaproteobacteria* genera [*Thorsellia* (89.9 %), an unclassified taxon in *Halomonadaceae* (2.4 *%*), an unclassified taxon in *Alteromondales* (2.1 %), and *Actinobacter* (0.9 %)] dominated the bacterial communities in *C. tarsalis* pupae (Additional file 6: Table S6)*.* However, the composition of the bacterial communities in the two samples varied considerably. Pupae collected from untreated control contained a greater percentage (93.2 % of 101,093 sequences) of *Thorsellia* and the bacterial community was similar to late-instar larvae. The bacterial community in pupae collected from the high *Bti* treatment contained a much smaller (1 % of 7,835) relative abundance of *Thorsellia* and was more similar to that in adults.

### Newly emerged non-blood-fed adult *C. tarsalis*

Overall, 277 genera representing 831 OTUs (47,700 sequences from five samples) were found in newly emerged non-blood-fed adult *C. tarsalis.* The most abundant bacterial phyla found in eclosing *C. tarsalis* included *Proteobacteria* (83.0 %), *Bacteroidetes* (8.2), *Actinobacteria* (4.0 %), *Firmicutes* (3.3 %), and *Cyanobacteria* (1.0 %) (Additional file 1: Table S1). Another 22 bacterial phyla contributed for < 1 % of the total sequences recovered from adults. At the family level, the bacterial communities of adult *C. tarsalis* were dominantly enriched with an unclassified taxon of *Halomonadaceae* (43 %), *Moraxellaceae* (10.9 %), *Commamonadaceae* (7.2 %), and *Flavobacteriaceae* (6.9 %) (Fig. 3). Three *Gammaproteobacteria* members [*Halomonas* (29.9 %), an unclassified taxon in *Altermonadales* (19 %), and *Acinetobacter* (10.4 %)] were the dominant genera found in adult mosquitoes (Additional file 5: Table S5). *Thorsellia* accounted for 1.3 % of all the sequences recovered from adult mosquitoes (Additional file 7: Table S7).

### Diversity of bacterial communities across developmental stages of *Culex*

**Fig. 5:**
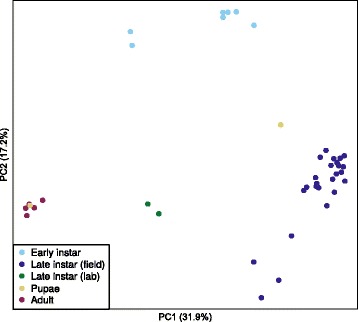
Principal coordinate analysis (based on Bray-Curtis distances) of bacterial communities in *C. tarsalis.* Bacterial communities in early and late instars, pupae, and adults sampled from outdoor mesocosms, and in laboratory-reared late larval instars of *C.tarsalis* were significantly separated. Pupae and adults were collected only from high *Bti* treatment and untreated control (see material and methods for details). Late instar larvae were sampled from all treatments

**Fig. 6:**
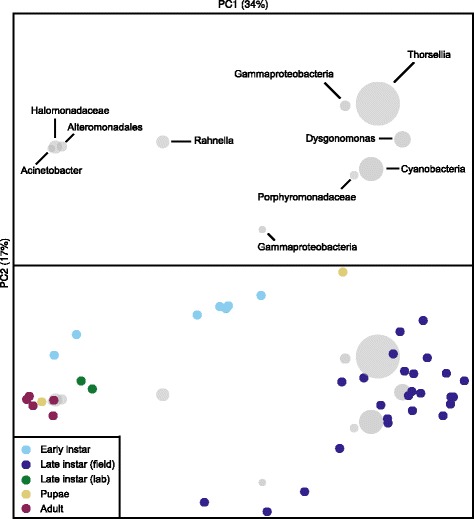
Principal coordinate analysis ordination (based on weighted UniFrac distances) of bacterial communities sampled from different stages of *C. tarsalis*. Top panel: The ten most abundant bacterial taxa found in the different mosquito life stages as they relate to the ordination space in the bottom panel. Bottom panel: Mosquito life stages projected on bacterial community profiles of samples collected in this study. “Lab” is late instar larvae from a laboratory colony of *C. tarsalis*

Beta diversity of bacterial communities differed significantly among mosquito developmental stages as assessed by multi-response permutation procedure (MRPP) on the Bray-Curtis and UniFrac distance matrices (Fig.s 5 and 6: MRPP analysis: *A*: 0.32, *p* < 0.001 for both). Bacterial diversity in early-instar *Culex* larvae from the first sampling date separated significantly from that of the late-instar larvae, pupae, and adults. Bacterial communities from lab-reared mosquitoes differed significantly from the bacterial communities in field-collected samples (Fig. 5). Only two samples of pupae and two samples of lab-reared larvae were analyzed in this study. Bacterial communities from one of the two pupal samples grouped tightly with adult samples, whereas the other sample was closely clustered to samples of field-collected late-instar larvae, mainly due to the differences in abundance of *Thorsellia* (Fig. 6). Bacterial communities from late-instar *Culex* larvae collected from the field were significantly separated from the other developmental stages (PC 1) and were dominated by *Thorsellia* and *Cyanobacteria*.

**Fig. 7:**
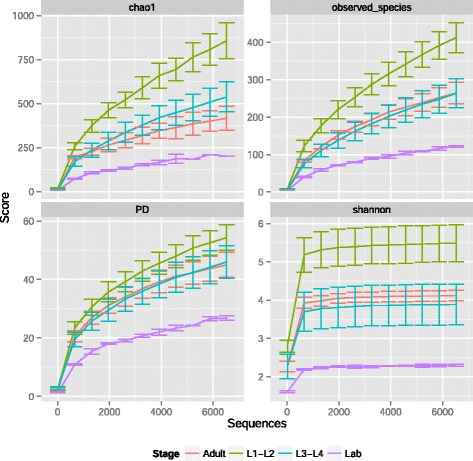
Alpha diversity. Alpha diversity (Chao1, Phylogenetic diversity, observed species and Shannon) of bacterial communities (based on OTUs) in different *Culex* mosquito stages: early-instar larvae, late-instar larvae (lab-reared or field-collected) and adults. Pupal samples were not included in the alpha diversity plots shown below because there was a large variation in the number of sequences between the two samples analyzed

Alpha diversity of bacterial communities in early instar *C. tarsalis* larvae was significantly higher than the bacterial diversity found in field-collected late larval instars and adults, and in laboratory-reared late-instar larvae (*p* < 0.001, Fig. 7). The diversity of bacterial communities in pupae varied considerably between the two samples and did not differ from the other developmental stages. The laboratory-reared larvae contained significantly less diverse bacterial communities than did larvae collected from the field (Fig. 7).

### Influences of *Bti* and habitat age on gut bacterial communities of late instar mosquitoes

**Fig. 8:**
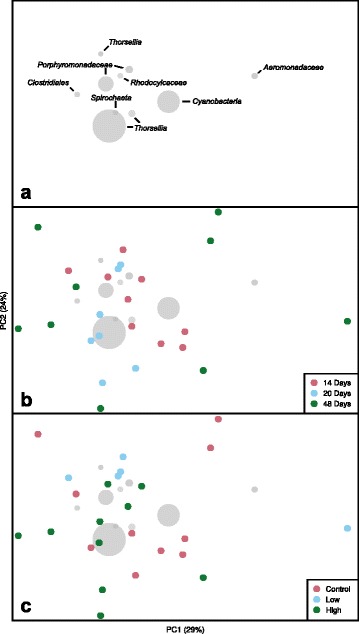
Principal coordinate analysis ordination (based on weighted UniFrac distances) of bacterial communities sampled from *C. tarsalis* late-instar larvae. Top panel (**a**): 10 most abundant taxa found in field-collected late-instar *C. tarsalis* larvae as they relate to the ordination space in the middle and bottom panels. Middle panel (**b**): bacterial community profiles from mosquitoes sampled in different habitat ages. Bottom panel (**c**): bacterial community profiles from mosquitoes sampled in three *Bti* treatments. Neither *Bti* treatment nor sampling date significantly influenced the bacterial communities in late-instar larvae of *C. tarsalis*

The bacterial communities in the late instar *Culex* larvae were not influenced significantly by age of the habitat or the larvicide treatments (Fig.s 8 and 9; MRPP A ≤ 0.1), although there was noticeable separation of bacterial communities from high *Bti* treatments from the other treatments. The bacterial communities in these larvae were dominated by OTUs of *Thorsellia*, *Cyanobacteria*, *Porphyromonadaceae*, *Aeromonadaceae*, *Rhodocyclaceae*, *Clostridiales* and *Spirochaeta* in descending order of abundance (Fig. 8).

**Fig. 9:**
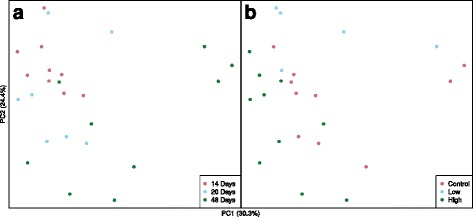
Principal coordinate analysis of bacterial communities in *C. tarsalis* late-instar larvae. Late-instar larvae collected from three *Bti* treatments (**a**), and three sampling dates (**b**)

## Discussion

Marker gene sequence analysis revealed the dynamics of bacterial community structure across developmental stages of *Culex tarsalis* (Fig. 1) and suggested transstadial transmission of some bacterial groups in *Culex* mosquitoes from North America. Overall, 235 bacterial OTUs in the phyla *Proteobacteria*, *Cyanobacteria*, *Bacteroidetes*, *Actinobacteria*, *Firmicutes*, *Spirochaetes*, and *RsaHF231* persisted from the early larval instars to newly emerged adults of *C. tarsalis*. The relative abundance of taxa in bacterial microbiome differed among the developmental stages (Fig. 5) but, within 3^rd^ and 4^th^ instars, did not differ significantly across sampling dates or in response to changes in the microbial community of the feeding zone that occurred following the application of a high dose of biopesticides. Our study provides additional evidence that, contrary to a commonly accepted hypothesis [17], metamorphosis does not completely eliminate gut microbiota. In *Culex pipiens* mosquitoes, some bacterial communities from the larval stages were shown to be sequestered in meconial peritrophic membrane and can be passed to the adult stages [17].

*Thorsellia* was found across all stages of *C. tarsalis* collected from the field but was rare in mosquitoes from laboratory colonies. However, the proportion of *Thorsellia* sequences in field-collected mosquitoes decreased during development. In adults derived from field-collected larvae, *Thorsellia* only accounted for 3.3 % of all sequences identified to the genus level, as compared to the early (31 %), and late (52.3 %) instar larvae, and pupae (93 %). This genus accounted for only 0.09 % of sequences identified to genus level from a lab colony of late *C. tarsalis* instar larvae suggesting that this bacterium is associated more strongly with natural aquatic habitats. The possible symbiotic role played by the *Thorsellia* to the different species of mosquitoes is currently unknown. Briones *et al*. reported a dominance of *T. anophelis* in the guts of adult *Anopheles gambiae* [27]. Likewise, Wang and colleagues found *Thorsellia* to be the dominating species in newly emerged adults making up 2/3 of their bacteria [19]. In this study, *Thorsellia* sequences obtained from *C. tarsalis* adults only accounted for a very small proportion of the bacterial sequences. That said*, Thorsellia* was found to be the primary resident in the guts of early and late larval instars of field-collected *C. tarsalis* (this study and 23). Overall, nearly 44 % of the sequences identified to genus level in the present study were *Thorsellia* sequences. Our finding indicates the persistence of taxa from this bacterial genus across all developmental stages of *C. tarsalis*.

Early instar larval mosquitoes harbored significantly more diverse bacterial communities (in three of four diversity indices) than the late-instar larvae collected from the field, and pupae and adult stages. The early instar larvae were sampled four days after the onset of the experiment and the bacterial communities in the guts of these mosquitoes mirrored the higher bacterial diversity observed in the water column on this sampling date [33]. It is unknown whether the reduction of bacterial communities especially during the late stages of development was due to the dominance of phytoplankton (particularly *Cyanobacteria*) in the water column (the feeding zone of *Culex* larvae) or the reduction of some bacterial taxa from the gut of the mosquitoes during metamorphosis. In addition, the early instar larvae likely included *Culex stigmatosoma* and *Culex quinquefasciatus,* and the greatest diversity observed in this stage might be because different mosquito species were present in the samples.

Late instar *Culex* larvae maintained fairly stable bacterial communities in their gut regardless of changes of microbial communities in the feeding zones of developmental sites. Duguma *et al.* also found that the bacterial communities within mosquito larvae did not change significantly during succession in two bioremediation treatments [23]. As revealed by PCoA ordinations, marked differences in the diversity of bacterial taxa were found between late instars, pupae and adults. Although the majority of the microbes found in the feeding stages (i.e., larvae) were lost likely during metamorphosis, our study suggests that certain species of bacteria were found among life stages investigated and warrant further experimental evaluation of these microbiomes whether they are obligate or facultative symbionts and persist through the different mosquito life stages.

The microbiome communities in laboratory-reared mosquitoes differed significantly from those found in mosquitoes in nature. We found that bacterial communities from field-collected *Culex* larval guts were significantly more diverse than their lab-reared counterparts. On average, 21 % of the sequences per sample from field-collected larvae were *Cyanobacteria,* whereas this taxon was virtually absent from the guts of lab-reared mosquitoes (Fig. 1). Similarly *Bacteroidetes* accounted 17 % of sequences found in the larvae collected from field whereas this bacterium only accounted 3 % of the sequences recovered from the lab-reared colony. The bacterial communities from laboratory-reared *C. tarsalis* larvae were primarily dominated by *Rahnella* (*Gammaproteobacteria: Enterobacteriaceae*), which occurred rarely in the field-collected larvae. Rani *et al*. also reported a significant reduction of bacterial diversity in lab-reared *Anopheles stephensi* [18]. It is well documented that gut microbial consortia play a significant role in shaping the physiology of insects [34, 35]. Bacteria found in lab-reared fruit flies were either rare or absent from wild *Drosophila* populations [22] posing a fundamental question on the extrapolations of many lab-reared host insect-microbiome models to the natural populations.

The high *Bti* treatment (equivalent to 48 kg Vectobac G ha^-1^) that reduced late (3^rd^ and 4^th^ instars) larval mosquito abundance by >50 % for a month, changed the microbiota (bacterial communities and phytoplankton) and nutrient concentrations in the water column [33]. However, neither the differences in planktonic bacterial communities caused by this larvicide treatment nor the differences in succession of bacterial communities across time influenced the bacterial communities in the gut of *C. tarsalis*. Interestingly, the water column bacterial diversity was significantly higher in the high *Bti* mesocosms and significantly separated from the other treatments on this date [33].

Although the majority of late instar larvae were removed from the water column by the high *Bti* treatment, the late instar larvae that were analyzed in this study were likely unaffected by the *Bti* application at the time of sampling. Nine weeks (44 days) after the *Bti* application, larval mosquito abundance in the high *Bti* treated mesocosms was significantly higher than in the low *Bti* and untreated control mesocosms [33]. Assessing the gut microbiota of mosquitoes surviving the biopesticide treatment has important implications because it might help understand the resistance mechanism and other physiological attributes provided by microbiota to the larval mosquitoes [35].

*C. tarsalis* is one of the most important vectors of arboviruses (e.g., western equine encephalomyelitis, St. Louis encephalitis and West Nile viruses) in North America. Interestingly, this mosquito species lacks endosymbionts such as *Wolbachia*, which is ubiquitous among several other arthropods including congeners [23,36]. Most other notable symbionts of mosquitoes such as *Asaia* and *Spiroplasma* are also lacking from *C. tarsalis*. The present study and a previous study [23] showed the dominance of the promising potential symbiont candidate, *Thorsellia*, in *C. tarsalis* collected from natural habitats. Both studies also reported the first evidence of this bacterium in a North American mosquito species. The function of this potential symbiont in the nutrition, physiology and vector competence of this mosquito species warrants further investigation.

## Conclusions

The metagenomic analysis of the bacteria microbiome (both cultivable and uncultivable) revealed significant differences among three life stages (larva, pupa and newly emerged adults) of *C. tarsalis* collected from the field, and larvae from a laboratory colony. The greatest diversity was observed in the early instar larvae and the lowest bacterial diversity was found in the lab colony. *Thorsellia* (*Gammaproteobacteria*) dominated the active feeding stages (larvae) collected from the field and persisted through non-feeding developmental stages suggesting, respectively, that these bacteria are ingested from the aquatic habitats and transstadial transmission is possible between stages. However, it is unknown whether the persistence of *Thorsellia* in the gut of larval mosquitoes is due to its resistance to digestion or is the result of continuous ingestion from the environment. The physiological role of this dominant bacterium within mosquitoes and whether it invades tissues also warrants further investigation.

The higher bacterial diversity in early instar larvae compared to the later developmental stages might have been due to either species-specific microbiomes that differed among *C. tarsalis* and two other congeners in the early -instar samples or other factors associated with the age of the habitat. The bacterial diversity was highest in the water column early in succession in the mesocosms. The gut bacterial community within late instar larval mosquitoes in outdoor mesocosms was conserved and did not change concomitantly with changes in the bacterial community present in the larval feeding zone across time as well as following the manipulation of larval density using a biopesticide treatment. The gut bacterial communities in pupae and adults were less diverse than in larvae. The mechanism(s) causing the loss of gut bacteria diversity during ontogeny require further study.

Finally, the bacterial communities found in laboratory-reared larvae were significantly less diverse compared to those found in field-collected larvae and only constituted a very small percentage (5 %) of the bacterial microbiomes found in the field-collected counterparts. *Thorsellia* was rare in mosquitoes from the laboratory colony. The low microbiome diversity found in the laboratory mosquitoes could have important implications for the interpretation of results and applicability to natural populations from mosquito-microbiomes studies using laboratory-reared mosquito larvae.

## Methods

### Study site and experimental design

The study was conducted in 1 m^2^ experimental outdoor mesocosms at the Aquatic and Vector Control Research Facility of the University of California Riverside Agricultural Experiment Station. Detailed description of the study site and experimental protocol are given in a previous study [33] but briefly, each of twelve mesocosms were filled with 300 liters of water from an irrigation reservoir and enriched with both organic (50 g of alfalfa rabbit pellets) and inorganic nutrients (40 g of ammonium sulfate) on September 28, 2012. The nutrients were added to stimulate mosquito and microbial colonization. On October 2, three treatments (two application rates of *Bti* [Vectobac G: high = 4.81 g/mesocosm and low = 0.06 g/mesocosm] and an untreated control) were assigned to the mesocosms in a completely randomized experimental design. The mean water level was adjusted to 30 cm on October 1, and adjusted again to 30 cm on October 5, and no water was added afterwards. The mean water level in the 12 mesocosms was 28 (±0.32; mean ± SE) cm on October 12, and 25 (±0.28) cm on October 19 and 17 (±0.65) cm on November 16.

### Mosquito sampling for bacterial DNA extraction

Five early instar *Culex* larvae per mesocosm were collected on October 1 (4 days after starting the experiment), placed in 95 % ethanol in 15 mL sterile centrifuge tubes and stored at -20 °C until DNA extraction. The identification of these early instars was not possible using morphology but late (3^rd^ and 4^th^) instar *Culex* larvae collected the following day was comprised of *C. stigmatosoma* (74 %), *C. quinquefasciatus* (23 %), and *C. tarsalis* (3 %). The proportion of *C. tarsalis* in the mesocosms increased over time while *C. stigmatosoma* tended to be more abundant early in experiment.

Five late (3^rd^ and 4^nd^) instar larvae of *C. tarsalis* larvae per mesocosm were sampled on three dates: October 12, October 18, and November 15 (i.e., 14, 20 and 48 days, respectively from the onset of the experiments) to determine the influences of *Bti* treatments and habitat age on bacterial community structure associated with *Culex* larvae. These dates correspond to 10, 16 and 44 days after *Bti* treatments, respectively. Two groups of five late instar *C. tarsalis* larvae from a laboratory colony were also sampled to compare bacterial communities of laboratory-reared mosquitoes with field-collected mosquitoes. *C. tarsalis* colonies have been maintained in the laboratory for > 5 years and the larvae were fed a mixture of yeast and ground rat chow [37].

For pupae and adult mosquitoes, late instar larvae were collected from the mesocosms, morphologically identified to *C. tarsalis* under a dissecting microscope, and then reared to pupae or to adulthood. Water from the corresponding mesocosms was used to rear the larvae. Immediately after pupation, two groups of five pupae from the high *Bti* treatment and the control were preserved in 95 % ethanol and placed in a -20 °C freezer until DNA extraction. The remaining pupae were then transferred to rearing cages and, immediately after emergence, five groups of five adult *C. tarsalis* (three from high *Bti* and two from control treatments) were placed in 95 % ethanol in 15 mL sterile tubes and kept in -20 °C freezer until DNA extraction.

### DNA extraction, PCR and Illumina library preparation

The procedures of DNA extraction and amplification of the V3 hypervariable region of 16S rRNA genes using polymerase chain reaction (PCR) were similar to those used for late larval instars in previous study [23]. In addition to late instar larvae, early (1^st^ and 2^nd^) instar larvae, pupae and adults of *C. tarsalis* were also sampled in the present study*.* All mosquito samples within the 15-mL tubes were sonicated for 3 min in iced sterile water according to a previous study [23]. DNA was extracted from only three (out of five) pooled, intact mosquitoes in 1.5 mL microcentrifuge tubes per replicate mesocosm using a Qiagen kit as described in [23]. PCR and Illumina library preparation were also carried out according to a previous study [23]. After libraries were prepared and quantified using an Agilent Bioanalyzer, all samples were normalized to 10 nM using Tris-HCl (10 mM, pH 8.5) and combined to create two multiplexed samples. The multiplexed samples were then subjected to a 2 × 150 base paired-end sequencing on a MiSeq Illumina platform at GENOSEQ (Sequencing and Genotyping Core) of the University of California Los Angeles, Los Angeles. Overall, a total of 41 mosquito samples [two late-instar samples from laboratory colonies, seven samples of field-collected early instars, 25 samples of late instars (taken on three sampling dates), two samples of pupae and five samples of adult mosquitoes] were submitted for sequencing.

### *Thorsellia* cloning and phylogeny

PCR in 25 μL reactions was performed with Illustra PuRe Taq Ready-To-Go PCR Beads (GE Healthcare, Uppsala, Sweden), 0.4 μM each of forward and reverse *Thorsellia* primers 207f (5’-GCACTAGGATGAACCCAGG-3’) and reverse primer 1277r (5’-CTTTATGAGTTCCGCTTACCC-3’), and 2 μL of DNA from larvae of *C. tarsalis*. These primers were designed for investigating *Thorsellia* in *Anopheles gambiae* sensu latu in a previous study [38]. The PCR program was 98 °C for 5 min followed by 30 cycles of [95 °C for 30 s, 55 °C for 30 s and 72 °C for 1 min] and a final step of 72 °C for 10 min. Amplification products (1.1 kb) were cloned into TOPO 2.1 (Invitrogen) and sequenced at Macrogen (South Korea).

Sequences were aligned with available GenBank accessions for three described *Thorsellia* species (*T. anophelis* [AY837748], *T. kanduguensis* [KM269289], and *T. kenyensis* [KM269290]) and a single outgroup taxon (*Arsenophonous nasoniae* [AY264674]) using MEGA version 6 [39]. Positions containing gaps or missing data were eliminated, resulting in a final dataset of 1070 positions. MEGA was again used to infer phylogentic relationships among the sequences using the Maximum Likelihood method based on the Tamura-Nei model [40]. The initial tree for the heuristic search was obtained automatically by applying Neighbor-Join and BioNJ algorithms to a matrix of pairwise distances estimated using the Maximum Composite Likelihood (MCL) approach, and then selecting the topology with superior log likelihood value. A discrete Gamma distribution was used to model evolutionary rate differences among sites [5 categories (+G, parameter = 0.05)].

### Sequence analysis, alignment, taxonomy assignment and statistical analysis

Analysis of the sequence reads was carried out using QIIME [41] version 1.7.0 and AXIOME version 1.6.0 [42] pipelines. Clustering of sequences to operational taxonomic units (OTUs) was carried out using cd-hit-est (multi-threaded version) with 97 % sequence identity [43]. Taxonomy assignment was conducted using the RDP classifier v2.2 with a confidence level of 0.6, and trained against the SILVA v111 16S/18S database [44]. All sequences that classified to *Eukaryota* were discarded. The statistical analyses of the sequences were carried out using procedures described in [33]. Briefly, beta diversity analysis using principal coordinate analysis (PCoA) based on Bray-Curtis dissimilarity distance matrix was carried out to assess the significance of differences among samples from different mosquito developmental stages, between *Bti* treatments and untreated controls, and among sampling dates. Beta diversity analyses were performed on OTU tables that were randomly subsampled (without replacement) down to the sample with the lowest number of sequences. Analyses including all stages were subsampled down to 6,539 sequences per sample, and analyses on late instar samples were subsampled down to 72,987 sequences per sample. The significance of the separation of sample groups in Bray-Curtis and UniFrac ordinations was assessed by MRPP [45] via QIIME. MRPP returns a within-group homogeneity value of *A* and a *p* value, which represents the probability of the observed differences between the groups occurring by chance. *A* values closer to 1 indicate increased sample similarity within-group, and an *A* value of 0 indicates the within-group similarity expected by chance. ‬ Alpha diversity measures based on phylogenetic distances were compared among samples within the mosquito developmental stages.

### Availability of supporting data

The data set supporting the results of this article is available in the ENA repository under project accession number PRJEB6788. Two representative clone sequences were deposited in GenBank under accession numbers KP215389 and KP215390. The data set supporting the results of this article are included within the article and its additional files.

## Additional files

Additional file 1:
**Table S1.** *Bacteria* phyla and percent (by number of sequences) abundance in *Culex* life cycle stages. Additional file 2:
**Table S2.** Shared bacterial OTUs across developmental *stages of C. tarsalis* collected from the field. Bacterial OTUs represented by at least 1 sequence per sample were included. Additional file 3:
**Table S3.** The most abundant bacterial OTUs (≥1 %) found in field-collected early-instar *Culex* larvae. Additional file 4:
**Table S4.** The most abundant bacterial OTUs (≥1 %) found in field-collected late-instar *C. tarsalis* larvae. Additional file 5:
**Table S5.**The most abundant bacterial OTUs (≥1 %) found in laboratory-reared late-instar *C. tarsalis* larvae. Additional file 6:
**Table S6.** The most abundant bacterial OTUs (≥0.5 %) found in *C. tarsalis* pupae (reared from late-instar larvae collected from the field). Additional file 7:
**Table S7.**The most abundant bacterial OTUs (≥1 %) found in *C. tarsalis* adults (reared from late-instar larvae collected from the field).

## References

[1] Merritt RW, Dadd RH, Walker ED (1992). Feeding behavior, natural food, and nutritional relationships of larval mosquitoes. Annu Rev Entomol.

[2] Clements AN (1992). *The biology of mosquitoes*: Development, nutrition and reproduction.

[3] Minard G, Mavingui P, Moro CV (2013). Diversity and function of bacterial microbiota in the mosquito holobiont. Parasit Vectors.

[4] Coon KL, Vogel KJ, Brown MR, Strand MR (2014). Mosquitoes rely on their gut microbiota for development. Mol Ecol.

[5] Pumpuni CB, Beier MS, Nataro JP, Guers LD, Davis JR (1993). *Plasmodium falciparum*: inhibition of sporogonic development in *Anopheles stephensi* by Gram-negative *Bacteria*. Exp Parasitol.

[6] Okech B, Gouagna L, Yan G, Githure J, Beier J (2007). Larval habitats of *Anopheles gambiae* s.s. (Diptera: Culicidae) influences vector competence to *Plasmodium falciparum* parasites. Malar J.

[7] Cirimotich CM, Ramirez JL, Dimopoulos G (2011). Native microbiota shape insect vector competence for human pathogens. Cell Host Microb.

[8] Takken W, Smallegange RC, Vigneau AJ, Johnston V, Brown M, Mordue-Luntz AJ, Billingsley PF (2013). Larval nutrition differentially affects adult fitness and *Plasmodium* development in the malaria vectors *Anopheles gambiae* and *Anopheles stephensi*. Parasit Vectors.

[9] Dong Y, Manfredini F, Dimopoulos G (2009). Implication of the mosquito midgut microbiota in the defense against malaria parasites. PLoS Patho.

[10] Ricci I, Valzano M, Ulissi U, Epis S, Cappelli A, Favia G (2012). Symbiotic control of mosquito borne disease. Patho Global Health.

[11] Bando H, Okado K, Guelbeogo W, Badolo A, Aonuma H, et al. Intra-specific diversity of *Serratia marcescens* in *Anopheles* mosquito midgut defines *Plasmodium* transmission capacity. Sci Rep. 2013;3. 10.1038/srep01641 PMC3622076 23571408

[12] Riehle MA, Jacobs-Lorena M (2005). Using bacteria to express and display anti-parasite molecules in mosquitoes: current and future strategies. Insect Biochem Mol Biol.

[13] Boissière A, Tchioffo MT, Bachar D, Abate L, Marie A (2012). Midgut microbiota of the malaria mosquito vector *Anopheles gambiae* and interactions with *Plasmodium falciparum* infection. PLoS Patho.

[14] Osei-Poku J, Mbogo C, Palmer W, Jiggins F (2012). Deep sequencing reveals extensive variation in the gut microbiota of wild mosquitoes from Kenya. Mol Ecol.

[15] Wang S, Ghosh AK, Bongio N, Stebbings KA, Lampe D, Jacobs-Lorena M (2012). Fighting malaria with engineered symbiotic bacteria from vector mosquitoes. Proc Natl Acad Sci USA.

[16] Caljon G, De Vooght L, Van den Abbeele J (2013). Options for the delivery of anti-pathogen molecules in arthropod vectors. J Invert Pathol.

[17] Moll RM, Romoser WS, Modrakowski MC, Moncayo AC, Lerdthusnee K (2001). Meconial peritrophic membranes and the fate of midgut bacteria during mosquito (Diptera: Culicidae) metamorphosis. J Med Entomol.

[18] Rani A, Sharma A, Rajagopal R, Adak T, Bhatnagar R (2009). Bacterial diversity analysis of larvae and adult midgut microflora using culture-dependent and culture-independent methods in lab-reared and field-collected *Anopheles stephensi*-an Asian malarial vector. BMC Microbiol.

[19] Wang Y, Gilbreath T, Kukutla P, Yan G, Xu J (2011). Dynamic gut microbiome across life history of the malaria mosquito *Anopheles gambiae* in Kenya. PLoS ONE.

[20] Chavshin AR, Oshaghi MA, Vatandoost H, Yakhchali B, Raeisi A, Zarenejad F (2013). *Escherichia coli* expressing a green fluorescent protein (GFP) in *Anopheles stephensi*: a preliminary model for paratransgenesis. Symbiosis.

[21] Chavshin AR, Oshaghi MA, Vatandoost H, Yakhchali B, Raeisi A, Zarenejad F, Terenius O (2015). Malpighian tubules are important determinants of *Pseudomonas* transstadial transmission and longtime persistence in *Anopheles stephensi*. Parasit Vectors.

[22] Chandler JA, Lang JM, Bhatnagar S, Eisen JA, Kop A (2011). Bacterial communities of diverse *Drosophila* species: ecological context of a host–microbe model system. PLoS Genet.

[23] Duguma D, Rugman-Jones P, Kaufman MG, Hall MW, Neufeld JD, Stouthamer R, Walton WE (2013). Bacterial communities associated with *Culex* mosquito larvae and two emergent aquatic plants of bioremediation importance. PLoS One.

[24] Lindh JM, Terenius O, Faye I (2005). 16S rRNA gene-based identification of midgut bacteria from field-caught *Anopheles gambiae sensu* lato and *A. funestus* mosquitoes reveals new species related to known insect symbionts. Appl Environ Microbiol.

[25] Kämpfer P, Lindh J, Terenius O, Haghdoost S, Falsen E, Busse HJ, Faye I (2006). *Thorsellia anophelis* gen nov, sp nov, a new member of the *Gammaproteobacteria*. Int J Syst Evol Microbiol.

[26] Kämpfer P, Glaeser SP, Nilsson LK, Eberhard T, Håkansson S, Guy L, Roos S, Busse H, Terenius O (2014). Proposal of *Thorsellia kenyensis* sp. nov. and *Thorsellia kandunguensis* sp. nov., isolated from the larvae of *Anopheles arabiensis* as members of the family *Thorselliaceae* fam. nov. Int J Syst Evol Microbiol.

[27] Briones A, Shililu J, Githure J, Novak R, Raskin L (2008). *Thorsellia anophelis* is the dominant bacterium in a Kenyan population of adult *Anopheles gambiae* mosquitoes. The ISME J.

[28] Chavshin AR, Oshaghi MA, Vatandoost H, Pourmand MR, Raeisi A, Terenius O (2014). Isolation and identification of culturable bacteria from wild *Anopheles culicifacies*, a first step in a paratransgenesis approach. Parasit Vector.

[29] Muturi EJ, Orindi BO, Kim CH (2013). Effect of leaf type and pesticide exposure on abundance of bacterial taxa in mosquito larval habitats. PLoS One.

[30] Kaufman MG, Chen S, Walker ED (2008). Leaf-associated bacterial and fungal taxa shifts in response to larvae of the tree hole mosquito, *Ochlerotatus triseriatus*. Microb Ecol.

[31] Xu Y, Chen S, Kaufman MG, Maknojia S, Bagdsarian M, Walker ED (2008). Bacterial community structure in treehole habitats of *Ochlerotatus triseiatus*: influences of larval feeding. J Am Mosq Control Assoc.

[32] Walker ED, Kaufman MG, Merritt RW (2010). An acute trophic cascade among microorganisms in the tree hole ecosystem following removal of omnivorous mosquito larvae. Community Ecol.

[33] Duguma D, Hall MW, Rugman-Jones P, Neufeld JD, Stouthamer R, Walton WE (2015). Microbial communities and nutrient dynamics in experimental microcosms are altered after application of a high dose of *Bti*. J Appl Ecol.

[34] Nkya TE, Akhouayri I, Kisinza W, David JP (2013). Impact of environment on mosquito response to pyrethroid insecticides: facts, evidences and prospects. Insect Biochem Mol Biol.

[35] Xia X, Zheng D, Zhong H, Qin B, Gurr GM (2013). DNA sequencing reveals the midgut microbiota of diamondback moth, *Plutella xylostella* (L.) and a possible relationship with insecticide resistance. PLoS One.

[36] Rasgon J, Scott T (2004). An initial survey for *Wolbachia* (*Rickettsiales*: *Rickettsiaceae*) infections in selected California mosquitoes (Diptera: Culicidae). J Med Entomol.

[37] Peck GW, Walton WE (2006). Effects of bacterial food quality and density on growth and whole body stoichiometry of *Culex quinquefasciatus* and *Culex tarsalis* (Diptera: Culicidae). J Med Entomol.

[38] Nilsson L: Isolation of Thorsellia from Kenyan Anopheles gambiae sensu lato and their breeding waters. MS thesis. Swedish University of Agricultural Sciences; 2012

[39] Tamura K, Stecher G, Peterson D, Filipski A, Kumar S (2013). MEGA6: Molecular Evolutionary Genetics Analysis version 6.0. Mol Biol Evol.

[40] Tamura K, Nei M (1993). Estimation of the number of nucleotide substitutions in the control region of mitochondrial DNA in humans and chimpanzees. Mol Biol Evol.

[41] Caporaso JG, Kuczynski J, Stombaugh J, Bittinger K, Bushman FD, Costello EK (2010). QIIME allows analysis of high-throughput community sequencing data. Nat Methods.

[42] Lynch MD, Masella AP, Hall MW, Bartram AK, Neufeld JD (2013). AXIOME: automated exploration of microbial diversity. GigaScience.

[43] Li W, Godzik A (2006). Cd-hit: a fast program for clustering and comparing large sets of protein or nucleotide sequences. Bioinformatics.

[44] Quast C, Pruesse E, Yilmaz P, Gerken J, Schweer T, Yarza P, Glöckner FO (2013). The SILVA ribosomal RNA gene database project: improved data processing and web-based tools. Nucl Acids Res.

[45] Mielke PW, Berry KJ, Johnson ES (1976). Multi-response permutation procedures for a priori classifications. Commun Stat Theor Methods.

